# Current status and issues regarding genetic medicine after cancer gene panel testing in Japanese patients with metastatic prostate cancer

**DOI:** 10.3892/mco.2025.2903

**Published:** 2025-10-09

**Authors:** Hideyasu Tsumura, Dai Koguchi, Ken-Ichi Tabata, Soichiro Shimura, Shuhei Hirano, Takefumi Satoh, Keiko Takahashi, Naomi Araki, Rika Kawata, Akinori Watanabe, Tsutomu Yoshida, Jiichiro Sasaki, Fumio Takada, Kazumasa Matsumoto

**Affiliations:** 1 Department of Urology, Kitasato University School of Medicine, Sagamihara, Kanagawa 252-0374, Japan; 2 Division of Genetics, Department of Genetics, Kitasato University Hospital, Sagamihara, Kanagawa 252-0375, Japan; 3 Department of Urology, Kitasato University Kitasato Institute Hospital, Tokyo 108-8642, Japan; 4 Department of Gastroenterology, Kitasato University School of Medicine, Sagamihara, Kanagawa 252-0374, Japan; 5 Division of Molecular Pathology, Department of Comprehensive Medicine, Research and Development Center for New Medical Frontiers, Kitasato University School of Medicine, Sagamihara, Kanagawa 252-0374, Japan; 6 Department of Research and Development Center for New Medical Frontiers, Kitasato University School of Medicine, Sagamihara, Kanagawa 252-0374, Japan; 7 Department of Medical Genetics and Genomics, Kitasato University Graduate School of Medical Sciences, Sagamihara, Kanagawa 252-0374, Japan

**Keywords:** comprehensive genomic profiling, genetic counseling, genetic testing, genotype-matched therapy, hereditary cancer, prostate cancer, secondary findings

## Abstract

The present study investigated the current status and issues regarding publicly reimbursed comprehensive genomic profiling (CGP) tests in Japan, as well as the implementation status of genetic counseling for cancer susceptibility genes identified by tumor-only sequencing in patients with prostate cancer. The data of 86 patients with metastatic prostate cancer who underwent CGP tests at a single institution between August 2019 and November 2024 were reviewed using either FoundationOne^®^ CDx (F1CDx; n=63) or FoundationOne^®^ Liquid CDx (F1LCDx; n=23) CGP tests. Of the 86 patients, 19 (22.1%) received genotype-matched therapy (GMT) under public health insurance (PHI) and 33 (38.4%) received subsequent systemic therapy (SST) because no GMT was identified or available under PHI or in clinical trials. Progression-free survival was significantly longer in the GMT/PHI group compared with the SST group (median, 6.5 vs. 3.5 months; P=0.006). Declines in serum prostate-specific antigen levels of ≥50% were more frequent in the GMT/PHI group [9/19 (47.3%)] than in the SST group [7/33 (21.2%)] (P=0.049). Of the 14 suspected carriers of deleterious germline alterations, 7 (50%) received genetic counseling; of which, 6/7 patients underwent confirmatory germline tests and 2 were diagnosed with hereditary breast and ovarian cancer. Cancer genetic medicine offers clinical benefits for patients with metastatic prostate cancer; however, most patients do not receive GMT after CGP testing. The implementation of genetic counseling for cancer susceptibility genes is thus insufficient and requires improvement.

## Introduction

Comprehensive cancer genome profiling (CGP) tests were approved by the public health insurance (PHI) system in Japan in June 2019, to identify genotype-matched therapies (GMTs) for patients with advanced solid tumors who fail to respond to standard therapies or for whom there are no appropriate standard therapies (1). Cancer genetic medicine is becoming part of routine clinical practice for patients with metastatic prostate cancer. The PROfound trial was conducted in patients with metastatic castration-resistant prostate cancer (mCRPC) who had deleterious alterations in prespecified genes related to homologous recombination repair (2). Olaparib was listed in Japanese PHI on December 2020 for patients with mCRPC with *BRCA1* or *BRCA2* deleterious alterations, confirmed by BRACAnalysis^®^ or CGP tests using FoundationOne^®^ CDx (F1CDx) or FoundationOne^®^ Liquid CDx (F1LCDx) CGP. The results of the PROpel and TALAPRO-2 trials were also approved by the Pharmaceuticals and Medical Devices Agency in Japan for patients with mCRPC with *BRCA* deleterious alterations (3,4).

The advent of cancer genetic medicine is an opportunity to recognize the importance of GMT for advanced prostate cancer and to validate the existence of hereditary cancers in patients with prostate cancer. When managing patients with metastatic prostate cancer, physicians must have the ability to interpret the results of CGP tests and identify appropriate GMTs for those patients. In addition, the need for genetic counseling (GC) has increasingly been recognized as the opportunity for CGP tests has increased in daily clinical practice.

In the present study, we investigated the status and issues regarding genetic medicine using publicly reimbursed CGP tests in patients with metastatic prostate cancer who failed to respond to standard therapies or for whom there were no appropriate standard therapies. We also investigated the status of GC and confirmatory germline testing for the secondary findings of cancer susceptibility genes identified by tumor-only sequencing.

## Materials and methods

### Patients and study design

This single-institution retrospective study evaluated the records of patients with metastatic prostate cancer who underwent CGP tests at Kitasato University Hospital (Kanagawa, Japan) between August 2019 and November 2024. Patients with metastatic prostate cancer who showed resistance to docetaxel or second-generation androgen receptor signaling inhibitors (ARSIs), including abiraterone, apalutamide, enzalutamide, and darolutamide, were subjected to CGP testing. Patients with androgen-indifferent prostate cancer, such as neuroendocrine prostate cancer, also underwent CGP testing. A total of 86 male patients were included, with a median age of 73.5 years (range, 46-85). High-volume disease was defined according to the CHAARTED criteria to evaluate the metastatic status (5).

Pathological slides for patients who underwent biopsy or radical prostatectomy at a facility other than our institution were reviewed by our institutional pathologists. Gleason scores were obtained from surgical specimens for patients who underwent radical prostatectomy, or from biopsy specimens at initial diagnosis for patients who did not undergo radical prostatectomy. All patients had histologically confirmed prostate cancer.

The Kitasato University Medical Ethics Organization approved this study (B21-110). All methods were carried out in accordance with relevant guidelines and regulations. Informed consent was obtained in the form of an opt-out option on the website and posters.

### CGP tests and actionable genomic alterations

F1CDx and F1LCDx were used for CGP tests in this study. F1CDx is a tissue-based hybrid capture next-generation sequencing (NGS) assay, and F1LCDx is a liquid biopsy-based NGS assay. Both tests analyze 324 cancer-related genes and selected intronic regions of 36 genes, designed to detect base substitutions, insertions/deletions, copy number alterations, gene rearrangements, microsatellite instability (MSI), and tumor mutation burden (TMB). Sequencing covers the entire coding regions of the included genes and selected intronic regions relevant for fusion detection (1). CGP testing was carried out using F1LCDx if the quality or quantity of an archival formalin-fixed paraffin-embedded tissue sample was inadequate for DNA analysis. CGP testing was only covered by Japanese PHI once in a person's lifetime at the time of this study, and individuals could thus not undergo both F1CDx and F1LCDx tests under PHI.

The evidence level was categorized as A to F based on the clinical practice guidelines for CGP tests in cancer diagnosis and treatment (Edition 2.0) issued by the Joint Consensus of Japanese Society of Medical Oncology, Japan Society of Clinical Oncology, and Japanese Cancer Association. When GMT was identified by CGP tests, actionable genomic alterations were defined as alterations at or above evidence level D (1,6,7).

### GC

If the patient wanted to know the results of the possible germline gene alterations detected by the CGP test, the physician in charge of genomic medicine disclosed if the patient had secondary findings of cancer susceptibility genes identified by tumor-only sequencing. The patients were advised to receive GC from the physician in charge of genomic medicine. If a patient was willing to receive GC, our hospital provided a system in which the patient called the Genetics Division and made an appointment. After receiving GC from clinical geneticists and certified genetic counselors, the patient then decided if they wanted to undergo confirmatory germline tests.

### Statistical analyses

Three different grouping strategies were employed in this study based on the analytical objectives. First, to evaluate clinical characteristics and the utility of testing modalities, patients were grouped according to the type of CGP test received: F1CDx and F1LCDx. Second, for the analyses of progression-free survival (PFS) and prostate-specific antigen (PSA) 50% decline, patients were stratified based on the type of post-CGP treatment: GMT/PHI group and subsequent systemic therapy (SST) group. Third, for the overall survival (OS) analysis, patients who received SST or transitioned to best supportive care (BSC) were categorized as the non-GMT group, and their outcomes were compared with those of the GMT/PHI group. SST was defined as any prostate cancer treatment administered after CGP testing that was not genomically matched therapy. CRPC, PSA progression, and radiographic progression were defined according to the Prostate Cancer Working Group 3. Radiographic progression was evaluated according to Response Evaluation Criteria in Solid Tumors (RECIST 1.1) on computed tomography scan or the 2 + 2 rule on bone scintigraphy (8,9). Clinical progression was defined as cancer pain requiring administration of opioids, the need to initiate subsequent therapy, radiation therapy, surgical intervention for complications due to tumor progression, or deterioration in Eastern Cooperative Oncology Group (ECOG) performance to ≥ grade 3. A castrate testosterone level was defined as ≤50 ng/dl.

PFS was defined as the time from initiation of GMT or SST to first occurrence of any progression or death from any cause, whichever occurred first. The events required to estimate PFS were PSA progression, radiographic progression, clinical progression, or any cause of death. Declines in serum PSA levels ≥50% were compared by χ^2^ test. OS was calculated from the date of genetic testing to last follow-up. One patient was confirmed as positive by BRACAnalysis^®^ and started olaparib before CGP testing, and was considered as receiving GMT in PHI. PFS and OS were calculated from the start date of olaparib administration and BRACAnalysis^®^ testing, respectively, and the best PSA response to olaparib was assessed in this patient.

PFS and OS were estimated by the Kaplan-Meier method and compared using the Gehan-Breslow-Wilcoxon test. Multivariate Cox proportional hazards regression models were performed to evaluate the factors influencing PFS. Differences were considered statistically significant at P<0.05. All reported P-values are two-sided. All analyses were performed using Stata version 15 (Stata Corp., College Station, TX, USA) and GraphPad Prism version 8 (GraphPad Software, Inc., La Jolla, CA, USA).

## Results

### Patient profiles

Table I summarizes the clinical characteristics of the 86 patients in the present study. To assess the patient characteristics and the impact of each testing modality on clinical utility, the patients were divided into two groups based on the type of CGP test: F1CDx (n=63) and F1LCDx (n=23). The median age of the overall population was 73.5 years, with 73 years for patients who underwent F1CDx and 74 years for those who underwent F1LCDx. The median time to CRPC for the overall population was 16 months, with 14.1 months for F1CDx and 24.4 months for F1LCDx. The detection of deleterious *BRCA* alteration for the overall population was 16.2% (14/86), with 22.2% (14/63) for F1CDx and 0% (0/23) for F1LCDx.

**Figure 1:**
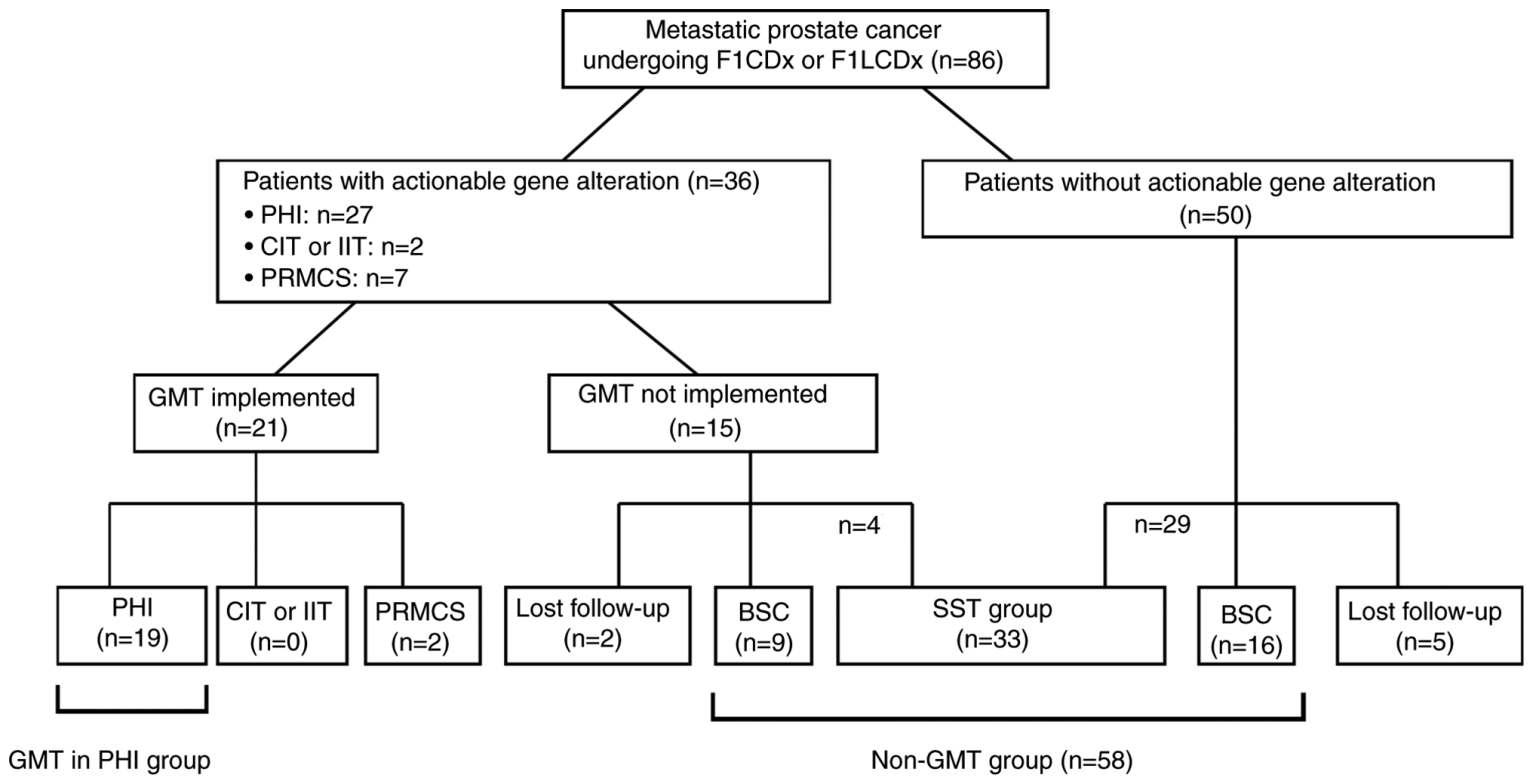
Patient profile after genetic testing in patients with metastatic prostate cancer. BSC, best supportive care; CIT, company-initiated trial; F1CDx, FoundationOne^®^ CDx cancer genome profiling; F1LCDx, FoundationOne^®^ Liquid CDx cancer genome profiling; GMT, genotype-matched therapy; PHI, public health insurance; PRMCS, patient-requested medical care system (phase II); IIT, investigator-initiated trial; SST, subsequent systemic therapy.

The patient profiles after CGP testing are shown in Fig. 1. Based on expert panel discussion, GMT was recommended in 36 patients (41.8%), including 27 in PHI, two in phase I or II clinical trials, and seven in patient-requested medical care system phase II (PRMCS). GMT was actually implemented in 21 patients (24.4%), including 19 (22.1%) in PHI, none in clinical trials, and two (2.3%) in PRMCS. Of the 36 patients for whom GMT was recommended, 15 (17.4%) did not receive the GMT due to deterioration of their general condition including prostate cancer death (n=9), ineligibility for clinical trial participation (n=1), the long distance to the clinical trial sites (n=1), unwilling to undergo the GMT (n=2), transfer to other institutions with loss to follow-up (n=2). Additionally, four patients received SST other than the recommended GMT.

Among the 50 (58.2%) patients in whom no actionable gene alteration was found, 29 received some kind of SST for prostate cancer. Sixteen patients (18.6%) switched to BSC due to the absence of aggressive prostate cancer treatment. Five patients (5.8%) lost to follow-up due to transfer to other institutions.

A total of 33 patients (38.4%) received some form of SST for prostate cancer other than GMT. Twenty-five patients (29.1%) switched to BSC due to the absence of aggressive prostate cancer treatment. Seven patients (8.1%) lost to follow-up.

### Oncological outcomes

**Figure 2:**
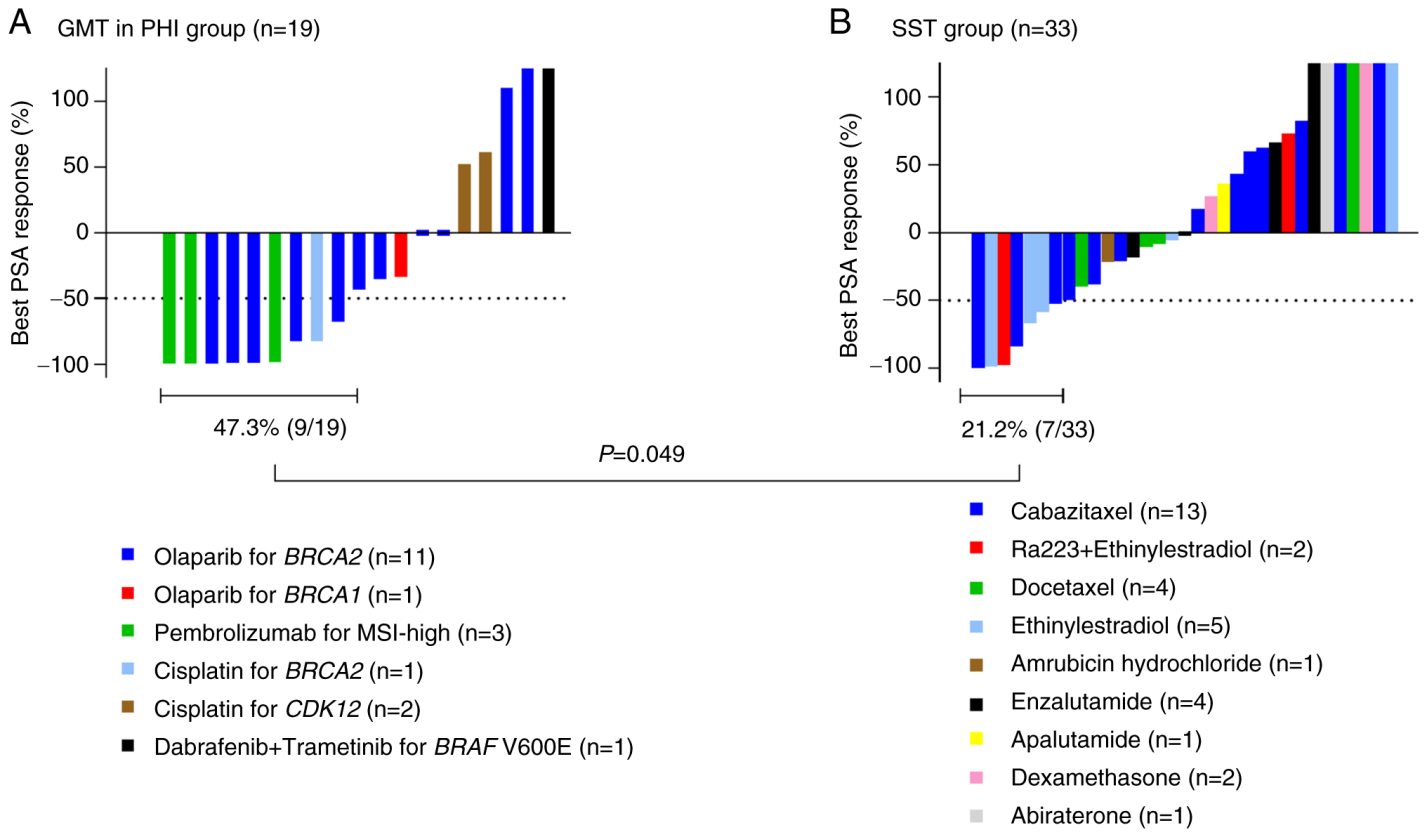
PSA response in (A) the GMT/PHI group, and (B) the SST group. GMT, genotype-matched therapy; PHI, public health insurance; MSI, microsatellite instability; PSA, prostate-specific antigen; SST, subsequent systemic therapy.

**Figure 3:**
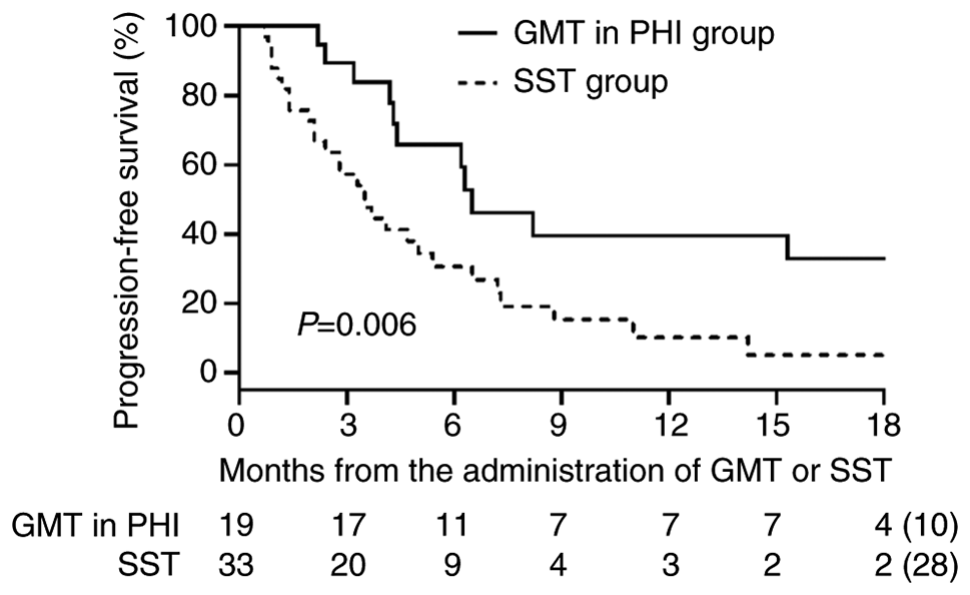
Progression-free survival in the GMT/PHI and SST groups. GMT, genotype-matched therapy; PHI, public health insurance; SST, subsequent systemic therapy.

**Figure 4:**
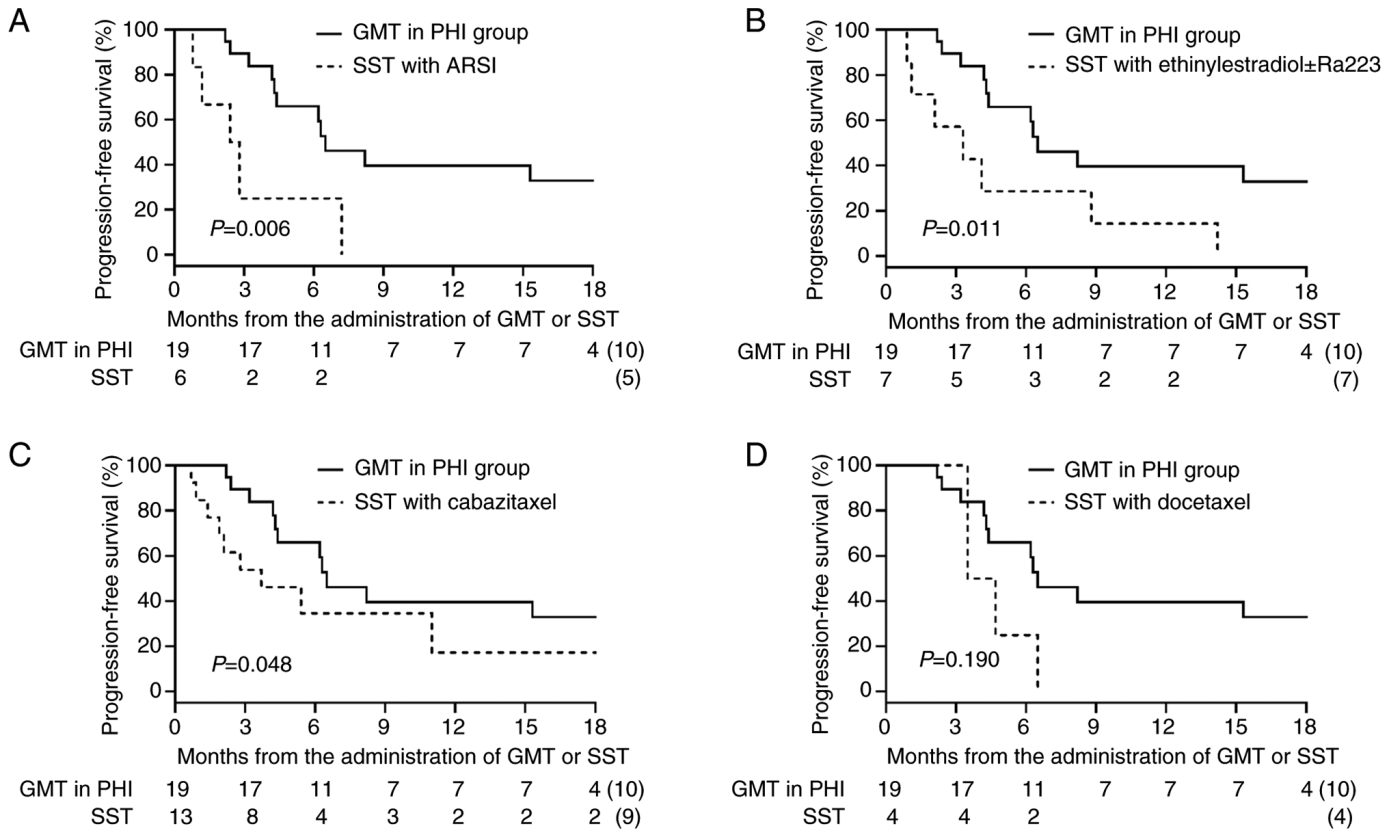
Comparison of progression-free survival between the GMT/PHI group and each SST subgroup with (A) ARSI rechallenge (n=6), (B) ethinylestradiol ± Ra223 (n=7), (C) cabazitaxel (n=13) and (D) docetaxel (n=4). ARSI, second-generation androgen receptor signaling inhibitor; GMT, genotype-matched therapy; PHI, public health insurance; SST, subsequent systemic therapy.

The PSA response in the GMT/PHI group and the SST group based on the results of CGP testing is shown in Fig. 2. Because individual data for GMT in PRMCS could not be disclosed, only data for GMT in PHI are presented in Fig. 2A. The treatments details for the 19 patients in the GMT/PHI group are also shown in Fig. 2A, the detailed oncological outcomes for the GMT/PHI group are shown in Table SI, and details of the treatments in the 33 patients in the SST group are shown in Fig. 2B. Declines in serum PSA levels ≥50% occurred more frequently in the GMT/PHI group (47.3%, 9/19) than in the SST group (21.2%, 7/33) (P=0.049). PFS in the GMT/PHI and SST groups is shown in Fig. 3. PFS was significantly longer in the GMT/PHI group than in the SST group (median: 6.5 vs. 3.5 months; P=0.006). We conducted a subgroup analysis for PFS in which each treatment regimen within the SST group was individually compared with the GMT/PHI group (Fig. 4). The median PFS in the GMT/PHI group was significantly longer than that in the SST subgroup treated with ARSI rechallenge (6.5 vs. 2.6 months; P=0.006), ethinylestradiol ± Ra223 (6.5 vs. 3.3 months; P=0.011), and cabazitaxel (6.5 vs. 3.7 months; P=0.048). In contrast, there was no statistically significant difference in PFS between the GMT/PHI group and the SST subgroup treated with docetaxel (6.5 vs. 4.1 months; P=0.190). After multivariate adjustment, receiving GMT/PHI was the only factor that showed a statistically significant association with longer PFS (hazard ratio 0.44, 95% confidence interval 0.20-0.93) (Table II).

**Figure 5:**
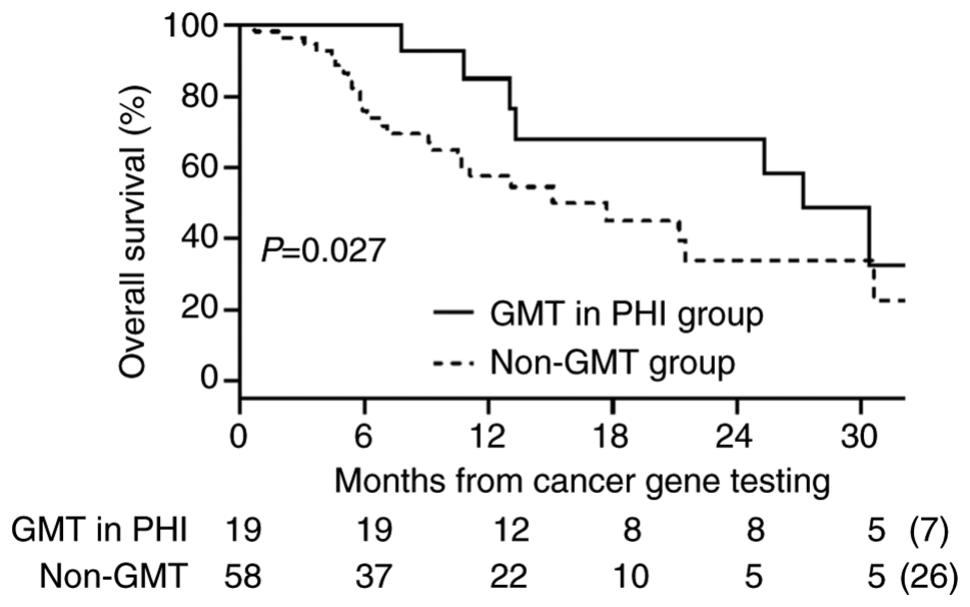
Overall survival in the GMT/PHI and non-GMT groups. GMT, genotype-matched therapy; PHI, public health insurance.

We defined the 25 patients who switched to BSC and the 33 patients who received SST as the non-GMT group (n=58). OS was significantly longer in the GMT/PHI group than in the non-GMT group (median: 27.2 vs. 17.7 months; P=0.027) (Fig. 5).

Gene abnormalities known to be involved in cancer, including alterations that may be targets of investigational therapies, were summarized in Table SII as potential actionable gene alterations identified by F1CDx and F1LCDx (https://doi.org/10.6084/m9.figshare.29923460).

### GC and confirmatory germline testing

Table III shows the implementation status of GC and confirmatory germline testing. Of the 14 patients suspected of carrying deleterious germline alterations, seven (50%) received GC, six of the seven patients received confirmatory germline tests and two were diagnosed with hereditary breast and ovarian cancer. Two patients underwent confirmatory genetic testing for Lynch syndrome, but the results were negative. The main motivation for receiving GC was the benefits for their family, but one patient visited the genetic division simply because his doctor told him to receive GC; however, this patient did not undergo confirmatory germline testing. Seven patients (50%) did not visit the genetic division because of poor health condition (n=1), death (n=1), not feeling the need (n=1), family opposition to GC (n=1), no relation to prostate cancer (n=1), and unknown reasons (n=2).

## Discussion

The current study included patients with metastatic prostate cancer who underwent F1CDx or F1LCDx testing after progression to an ARSI or docetaxel, or patients for whom there was no appropriate standard therapy due to androgen-indifferent prostate cancer. Outcomes in terms of PFS and declines in serum PSA levels ≥50% were significantly better in the GMT/PHI group compared with the SST group. While approximately one in five of these patients received GMT as a result of CGP testing, many patients who underwent CGP tests were unable to receive GMT, highlighting the need to address this issue in the future.

GMT was implemented in 21 patients (24.4%), including 19 (22.1%) in PHI and two (2.3%) in PRMCS, but a total of 33 patients (38.4%) received some form of SST other than GMT, because no actionable gene alterations were detected, or if gene alterations were detected, the patients were unable to access GMT. The timing of CGP testing is sometimes delayed in Japan since the CGP testing is limited to patients with mCRPC who developed resistance to docetaxel or ARSIs. As a result, patients experienced disease progression at the time of disclosure of CGP results and were no longer able to receive the recommended GMT. In addition, accessibility to GMT through clinical trials is also one of the issues that should be addressed. Some patients were unable to receive treatment because of a lack of accurate information regarding the eligibility criteria for clinical trials and the long distance to the clinical trial sites. Sharing clinical trial information among the hospitals and expanding clinical trial sites across Japan are necessary (1).

CGP testing can detect gene alterations and genomic signatures, including MSI, TMB, genome-wide loss of heterozygosity, and homologous recombination deficiency. Although several prospective randomized phase II trials have investigated the efficacy of CGP testing for solid tumors, the clinical benefit of GMT using CGP testing remains debatable (10-12). The SHIVA trial was conducted for patients with any kind of metastatic solid tumor refractory to standard of care. The use of GMT outside their indications does not improve PFS compared with treatment at physician's choice (10). The CUPISCO trial compared the efficacy of GMT vs. standard platinum-based chemotherapy in patients with an unfavorable cancer subset of unknown primary. Subgroup analysis showed that patients with an actionable molecular profile treated with GMT had longer PFS than those with an actionable molecular profile treated with standard platinum-based chemotherapy (hazard ratio 0.76, 95% confidence interval 0.42-0.99), with the greatest differences in patients treated with atezolizumab for TMB-high or MSI-high, vemurafenib plus cobimetinib for *BRAF* V600 or K601E alterations, or pemigatinib for *FGFR1*, *FGFR2*, or *FGFR3* alterations (12). These results support the effectiveness of CGP testing for patients for whom there is no appropriate standard treatment.

A previous study reported that *BRCA* deleterious alterations were detected by tumor genetic testing in approximately 10% of patients with mCRPC (2-4,13), which was lower than the 16.2% in the current study. Mateo *et al* (14) reported that *BRCA2* deleterious alterations were significantly more common in patients who developed mCRPC in a short period, and patients with *BRCA2* deleterious alterations had the lower time to androgen-deprivation therapy (ADT) progression compared to those without *BRCA2* deleterious alterations. Thus, this difference may be due to the relatively short time to CRPC observed in our patients undergoing CGP tests. In the present study, olaparib was administered to patients with mCRPC who were resistant to at least one kind of ARSI, with a similar treatment population to the PROfound study (2). Three of 12 patients receiving olaparib responded without progression for >24 months, confirming the existence of deep and durable responders among patients with *BRCA* deleterious alterations, after resistance to ARSI. Several potential biological mechanisms have been reported to underly long-term responses to poly ADP ribose polymerase (PARP) inhibitors. *BRCA* structural variants, such as rearrangements and homozygous deletions, are inherently resistant to reversion mutations and thus contribute to durable responses to PARP inhibitors (15). Buonaiuto *et al* (16) reported that the efficacy of PARP inhibitor is independent by *BRCA* domain defects or mutation types. These findings are partly consistent with our results. However, they are not sufficient to fully explain the mechanisms underlying long-term responses to PARP inhibitors. The underlying biological mechanisms need to be further explored.

The detection rate of MSI-high in prostate cancer is approximately 3%, and a PSA response ≥50% with immune checkpoint inhibitor (ICI) therapy has been reported to be 44-65% (17-19). Three patients with both MSI-high and TMB-high genomic signatures received pembrolizumab, all of whom showed a PSA response ≥50%, while two achieved undetectable PSA levels in the present study. Notably, in the two patients who achieved undetectable PSA levels, the dominant lesions were confined to the lymph nodes and the primary tumor. In contrast, one patient who died of prostate cancer had extensive bone metastases. The efficacy of ICIs may differ depending on the site of metastasis. In non-small cell lung cancer, lymph node and primary lung lesions tend to show higher response rates and better disease control with ICIs, whereas bone metastases are consistently associated with poor responses and worse survival outcomes (20,21). This likely reflects differences in the tumor microenvironment (22). In line with these findings, the differential response patterns observed in our study may reflect the underlying immune contexture of metastatic sites.

No patients in our study were identified as TMB-high and microsatellite stable (MSS), in contrast to a previous study that reported TMB-high with MSS in 0.7% of patients with prostate cancer. Although the scientific rationale for ICIs is similar between MSI-high and TMB-high with MSS, patients with prostate cancer with TMB-high plus MSS did not demonstrate the same durability to ICI treatment compared with MSI-high patients (17).

*BRAF* gain-of-function alteration, *RET* fusion, and *NTRK* fusion are rare alterations detected by CGP testing in patients with prostate cancer. The use of molecular targeted therapies, such as tumor-agnostic treatments, is approved for these genetic alterations under PHI in Japan (23-26). In the present study, we administered combination therapy with dabrafenib and trametinib in a patient with a *BRAF* V600E alteration. In this case, chest-to-pelvis computed tomography scans revealed no primary tumors other than prostate cancer, while an additional colonoscopy examination found no colorectal cancer and a dermatological evaluation found no evidence of malignant melanoma. In addition, F1LCDx identified the *AR* T878A alteration as a covariate, with a variant allele frequency of 3.5%. We observed a partial response in liver metastases on radiographic imaging. To the best of our knowledge, this is the first reported case of the efficacy of this combination therapy in a patient with *BRAF* Class I alterations, which occur at the V600 codon and exhibit extremely strong kinase activity by stimulating monomeric activation of *BRAF* in prostate cancer (27). There have been no case reports of successful treatment of prostate cancer with *RET* fusion and *NTRK* fusion to date.

CGP tests increase the opportunities for GC by identifying a proportion of patients with potential hereditary cancers caused by cancer susceptibility genes (28). The introduction of the CGP test for cancer treatment has changed physicians' awareness of hereditary cancers; CGP tests can not only find eligible patients for GMT, but can also reveal a predisposition to hereditary cancers. GC and confirmatory germline testing play a crucial role in connecting the early detection and early treatment of hereditary cancers in relatives (29-32). For metastatic prostate cancer, 11.8% of cases are reported to be hereditary cancers associated with single DNA-repair gene mutations (33); however, the current study only identified two cases of hereditary cancers through GC. The implementation status of GC after tumor-only sequencing at our institution was insufficient to identify germline carriers of cancer susceptibility genes (1,34). When patients struggle with cancer treatment, it is sometimes difficult for them to address secondary findings related to hereditary cancers, because the GC is not related to their cancer treatment. It may be difficult for patients to receive GC if they need to return to the hospital to receive it, given that most patients undergoing CGP tests are already spending a lot of time on their cancer treatment. Considering the backgrounds of these patients, our system in which patients were required to make their own appointments for GC may have resulted in them missing opportunities to receive GC. To improve the implementation of GC, Kikuchi *et al* (1) reported that a system in which GC was provided on the same day as the disclosure of CGP results led to an increase in the number of patients receiving GC. At our institution, there is a shortage of certified genetic counselors and clinical geneticists who can address the secondary findings generated by CGP testing. Increasing the availability of these professionals would help ensure that GC can be provided on the same day as the disclosure of CGP results. In addition, there remains a lack of awareness among patients and their families regarding hereditary cancers. This highlights the need for broader educational efforts to improve understanding of genetic risk.

Our study had several potential limitations, including its retrospective nature. First, this was a single-institution study, and the results thus lack adequate power to make generalizations. Treatment strategies vary among physicians, and these differences may have influenced our results. Second, olaparib was not used in combination with an ARSI. The results of the PROpel and TALAPRO-2 trials were recently approved by the Pharmaceuticals and Medical Devices Agency in Japan (3,4). Combined therapy with a PARP inhibitor and an ARSI showed clinically meaningful improvement as a first-line therapy in patients with mCRPC with *BRCA* deleterious alterations who showed resistance to ADT alone or ADT plus traditional hormonal therapy. The present study did not reflect these new prospects for PARP inhibitors for mCRPC. Third, a multivariate Cox proportional hazards analysis was performed to adjust for baseline characteristics in the analysis for PFS. However, we could not fully eliminate all potential confounders such as age, ECOG performance status, treatment lines, and tumor burden. These confounders are likely to influence the interpretation of our results. Future studies with better control of these variables are warranted to validate our findings. Fourth, the small number of patients and short follow-up period may have been insufficient to address the efficacy of genetic medicine and further studies with longer follow-up and data from multiple institutions involving more patients are needed. Although this study included a small number of cases and the experience was limited, GMT based on the results of CGP tests has had an impact on our daily clinical practice.

In conclusion, CGP testing offers new hope for patients with metastatic prostate cancer for whom there is currently no appropriate subsequent therapy. However, most patients do not receive GMT in our daily practice. The implementation of GC for cancer susceptibility genes is thus insufficient and requires improvement. Further developments in CGP testing are needed to improve the clinical outcomes, including the development of novel GMTs and the optimization of timings for CGP testing.

## Supplementary Material

Oncological outcomes for GMT in PHI group (n=19).

Summary of potential actionable gene alterations.

## Figures and Tables

**Table I:** Patient characteristics.

Variable	F1CDx (n=63)	F1LCDx (n=23)	Total (n=86)
Median age (range), years	73 (46-85)	74 (57-84)	73.5 (46-85)
Median PSA at initial diagnosis (range), ng/ml	95.5 (1.13-9,747)	21.0 (6.86-8,537)	55.0 (1.13-9,747)
Metachronous (TxNxM0)	21 (33.3)	14 (60.9)	35 (40.6)
Radical prostatectomy	10 (15.9)	5 (21.7)	15 (17.4)
Prostate radiotherapy	9 (14.3)	8 (34.8)	17 (19.8)
Synchronous (TxNxM1)	42 (66.7)	9 (39.1)	51 (59.4)
High volume (CHAARTED criteria)	31 (49.2)	8 (34.7)	39 (45.3)
Gleason score			
7	5 (7.9)	2 (8.7)	7 (8.1)
8	16 (25.4)	8 (34.8)	24 (27.9)
9 or 10	42 (66.7)	12 (52.2)	54 (62.8)
Unknown	0 (0.0)	1 (4.3)	1 (1.2)
Median time from initiation of ADT to CGP tests (range), months	32.8 (0.5-238.5)	73.3 (24.7-151.4)	40.6 (0.5-238.5)
Median time to CRPC (range), months	14.1 (1.9-98.6)	24.4 (1.4-88.2)	16.0 (1.4-98.6)
Deleterious *BRCA* alteration	14 (22.2)	0 (0)	14 (16.2)
*BRCA1*	1	0	1
*BRCA2*	13	0	13
MSI-high and TMB-high	3 (4.8)	0 (0.0)	3 (3.5)
*BRAF* V600	0 (0.0)	1 (4.3)	1 (1.2)

Data are presented as n (%) unless otherwise indicated. ADT, androgen-deprivation therapy; CGP, comprehensive cancer genomic profiling; CRPC, castration-resistant prostate cancer; F1CDx, FoundationOne^®^ CDx cancer genome profiling; F1LCDx, FoundationOne^®^ Liquid CDx cancer genome profiling; MSI, microsatellite instability; PSA, prostate-specific antigen; TMB, tumor mutation burden.

**Table II:** Multivariate analyses using a Cox proportional hazards model for progression-free survival.

Variable	HR	95% CI	P-value
Baseline PSA at initiation of GMT or SST, continuous	1.00	0.99-1.00	0.64
CHAARTED criteria, high volume vs. low volume	0.97	0.50-1.89	0.94
Time to CRPC, continuous	1.00	0.98-1.02	0.85
GMT/PHI group vs. SST group	0.44	0.20-0.93	0.03

CI, confidence interval; CRPC, castration-resistant prostate cancer; GMT, genotype-matched therapy; HR, hazard ratio; PHI, public health insurance; PSA, prostate-specific antigen; SST, subsequent systemic therapy.

**Table III:** Genetic counseling and confirmatory germline testing for cancer susceptibility genes.

Alteration	Age, years	VAF, %	GC	Reasons for receiving or not receiving GC	Germline testing	Germline alteration	Family history of cancer
*BRCA2* (A902fs*2)	Mid 40s	64.4	Received	Told by doctor to receive GC	Untested	Unknown	Breast, mother; stomach, father; uterus, grandmother
*BRCA2* (V1447fs*1)	Early 70s	83.6	Received	For his family	Tested	Yes	None
*BRCA2* (HD exon 9-27)	Early 70s	-	Received	For his family	Tested	No	Prostate, father and uncle; stomach, grandmother
*BRCA2* (E2877*)	Early 50s	76.9	Received	For his family	Tested	Yes	Brain, aunt
*BRCA2* M2393fs*19	Early 80s	36.6	Received	For his family	Tested	No	Lung, father
MSI-high	Early 70s	-	Received	For his family	Tested	No	None
MSI-high	Mid 70s	-	Received	For his family	Tested	No	Tongue, sister
*BRCA1* (HD, exon 5-8)	Mid 50s	-	Not received	Poor health condition	Untested	Unknown	None
*BRCA2* E790fs*20	Early 80s	34.5	Not received	Death	Untested	Unknown	None
*BRCA2* (I729fs*21)	Late 70s	25.7	Not received	Not feeling the need	Untested	Unknown	Uterus, grandmother
*BRCA2* (c.316+1G>T)	Mid 80s	31.9	Not received	Family opposition	Untested	Unknown	Colon, father; breast, aunt
*WT1* c.1001+1G>T	Early 70s	49.8	Not received	No relation to prostate cancer	Untested	Unknown	Biliary tract, sister; stomach, sister; breast, sister; colon, cousin
*ATM* c.5918+1G>T	Mid 70s	71.4	Not received	Unknown	Untested	Unknown	Pancreas, father; stomach, uncle
*PMS2* (E836fs*15)	Late 60s	85.7	Not received	Unknown	Untested	Unknown	None
*MSH2* (Q170*)		66.3					

GC, genetic counseling; MSI, microsatellite instability; VAF, variant allele frequency.

## Data Availability

The data generated in the present study may be requested from the corresponding author.
