# Supplementary material for: Current status and issues regarding genetic medicine after cancer gene panel testing in Japanese patients with metastatic prostate cancer
Source: Mol Clin Oncol. 2025 Oct 9;23(6):108. doi: 10.3892/mco.2025.2903 (PMC12557695; doi:10.3892/mco.2025.2903)
Supplement: Oncological outcomes for GMT in PHI group (n=19). [file Supplementary_Data1.pdf]

Table SI. Oncological outcomes for GMT in PHI group (n=19).

Gene	GMT	Treatment lines	F1LCDx	Best PSA response, %	Type of progression	PFS, months	Status
MSI-high & TMB-high (25.34 Muts/Mbp)	Pembrolizumab	1	-	-99.9	-	16.1	Ongoing
MSI-high & TMB-high (103.68 Muts/Mbp)	Pembrolizumab	5	Yes	-99.8	-	2.6	Ongoing
BRCA2 (N863fs*18)	Olaparib	1	-	-99.5	Radiographic	15.3	Death
BRCA2 (M2393fs*19)	Olaparib	3	-	-99.3	-	29.1	Ongoing
BRCA2 (HD)	Olaparib	3	-	-98.9	-	17.0	Ongoing
MSI-high & TMB-high (17.7 Muts/Mbp)	Pembrolizumab	3	-	-98.4	Radiographic	6.3	Death
BRCA2 (I605fs*11)	Olaparib	1	-	-82.9	-	2.5	Ongoing
BRCA2 (A902fs*2)	Cisplatin	4	-	-82.4	Clinical (drug change)	6.2	Death
BRCA2 (I729fs*21)	Olaparib	3	-	-67.7	-	37.7	Ongoing
BRCA2 (c.316+1G>T)	Olaparib	2	-	-43.2	-	5.8	Ongoing
BRCA2 (E2877*)	Olaparib	2	-	-35.5	-	3.7	Ongoing
BRCA1 (HD, exon 5-8)	Olaparib	3	-	-33.8	Clinical (opioid use)	4.3	Death
BRCA2 (Rearrangement exon 20)	Olaparib	2	-	0	-	26.8	Ongoing
BRCA2 (V1447fs*1)	Olaparib	2	-	0	Clinical (PS deterioration)	4.4	BSC
CDK12 (Q937fs*18)	Cisplatin	3	-	52	Clinical (PS deterioration)	2.4	BSC
CDK12 (Q780*)	Cisplatin	3	Yes	61	Clinical (PS deterioration)	4.2	Death
BRCA2 (HD)	Olaparib	3	-	110	Clinical (drug change)	3.2	BSC
BRCA2 (fusion, *BRCA2-STARD13*)	Olaparib	2	-	162	Clinical (PS deterioration)	2.2	Death
BRAF (V600E)	Dabrafenib + Trametinib	4	Yes	511	Radiographic	8.2	Ongoing

BSC, best supportive care; F1LCDx, FoundationOne® Liquid CDx cancer genome profiling; GMT, genotype-matched therapy; HD, homozygous deletion; MSI, microsatellite instability; PFS, progression-free survival; PHI, public health insurance; PS, performance status; PSA, prostate-specific antigen; TMB, tumor mutation burden.
